# ADAMTS-5 Decreases in Aortas and Plasma From Aortic Dissection Patients and Alleviates Angiotensin II-Induced Smooth Muscle-Cell Apoptosis

**DOI:** 10.3389/fcvm.2020.00136

**Published:** 2020-08-14

**Authors:** Tao Zeng, Jianting Gan, Yu Liu, Lei Shi, Zhengde Lu, Yan Xue, Rixin Xiong, Ling Liu, Zicong Yang, Yingzhong Lin, Jun Yuan

**Affiliations:** Department of Cardiology, The People's Hospital of Guangxi Zhuang Autonomous Region, Nanning, China

**Keywords:** acute aortic dissection, ADAMTS-5, extracellular matrix, smooth muscle cells, matrix metalloproteinase, apoptosis

## Abstract

**Background:** Acute aortic dissection (AAD) is associated with degeneration of the aortic media and accompanied by vascular extracellular matrix (ECM) remodeling. Recently, a disintegrin and metalloproteinase with thrombospondin type 1 motifs-5 (ADAMTS-5) has been reported to be involved in ECM remodeling and vascular diseases. The aim of this study was to examine ADAMTS-5 levels in AAD patients and investigate the underlying mechanisms.

**Methods:** Aortic tissue samples were collected from normal donors and AAD patients, and the expression of ADAMTS-5 was analyzed in all aortic tissues. In addition, plasma levels of ADAMTS-5, matrix metalloproteinase (MMP)-2 and MMP-9, and tumor necrosis factor-α (TNF-α) were measured in repeated samples from AAD patients and compared to the non-AAD (NAD) group. In addition, we investigated the effects of ADAMTS-5 in smooth muscle cell (SMC) apoptosis.

**Results:** The results showed that ADAMTS-5 expression was significantly reduced in the aortas of AAD patients and that SMCs were the main source of ADAMTS-5. In addition, the plasma ADAMTS-5 level was lower, but plasma MMP-2, MMP-9, and TNF-α levels were increased in the AAD patients. Multivariate linear regression analyses showed that a decreased ADAMTS-5 level in patients was independently associated with an increased risk of AAD. Furthermore, recombinant human ADAMTS-5 significantly ameliorated angiotensin (Ang II)-evoked SMC apoptosis.

**Conclusions:** ADAMTS-5 shows promise as a novel potential biomarker for AAD, and regulation of SMC is a possible mechanism for the effects of ADAMTS-5.

## Introduction

Acute aortic dissection (AAD) is a life-threatening cardiovascular disease associated with high rates of mortality and morbidity. AAD events are initiated by a circumferential or transverse tear of the intima, followed by rapid leakage of blood into the artery wall, which undergoes thrombosis and rupture of the aorta (1, 2). AADs can be classified according to origin of the intimal tear and/or involvement of the ascending aorta. Stanford type A dissections involve the ascending aorta and are treated via emergency surgical repair, whereas type B dissections only involve the descending aorta and usually require endovascular repair and/or medical therapy. Previous studies have indicated that if left untreated, ~75% of type A patients might die within 2 weeks following symptom onset (3). Therefore, immediate early diagnosis and treatment is crucial for improved survival.

The precise mechanisms underlying AAD have not been completely elucidated. Remodeling of extracellular matrix (ECM), decreases in smooth muscle cells (SMCs), and infiltration of inflammatory cells are thought to weaken the arterial wall and increase the risk of AAD (1). Histologically, compromised aortic integrity is the result of ECM remodeling within the aorta, including collagen degradation, elastic fiber fragmentation, and medial layer degeneration (4, 5). At the molecular level, diseased aortas present SMC apoptosis and activation of inflammation (5, 6). Loss of SMCs accelerates ECM remodeling by altering the production of metalloproteinases and proteoglycans, followed by weakening of the aortic wall (7, 8). Persistence of aortic inflammation also contributes to the apoptosis of SMCs along with medial degradation and ultimately increases the risk of intimal disruption (6, 9).

A disintegrin and metalloproteinase with thrombospondin motifs-5 (ADAMTS-5) is an ECM-degrading enzyme that is involved in ECM remodeling by degrading proteoglycans, such as aggrecan, versican, brevican, and neurocan (10, 11). Previous studies showed that ADAMTS-5 is reduced in coronary arteries and plasma from patients with coronary artery disease (12). Cikach et al. reported that ADAMTS-5 mRNA levels are significantly reduced in human AAD aortas, while other ADAMTSs are not altered (13). However, a report directly addressing circulating ADAMTS-5 levels in AAD patients is still lacking. Thus, the purpose of this study was to determine the expression and source of ADAMTS-5 in aortic tissue and plasma obtained from patients with AAD.

## Methods

### Collection of Human Aortic Tissue Samples

Aortic tissues (*n* = 9) were obtained from AAD patients who underwent emergency thoracic aorta replacement surgery. The control samples (*n* = 7) were obtained from heart donors who had suffered traffic accidents and were declared brain dead. The donors had no history of cardiovascular disease, and the aortic tissues showed no signs of pathology. All participants in this research underwent a standard clinical examination. The patients and donor families provided informed consent for donation. The study procedure was approved by the Medical Ethics Committee of the People's Hospital of Guangxi Zhuang Autonomous Region.

### Western Blotting

Total proteins were homogenized in RIPA lysis buffer, and protein concentrations were determined. Total proteins were separated via SDS-PAGE and then transferred to a PVDF membrane (Millipore, USA). The membranes were subsequently blocked with 5% skim milk in Tris-buffered saline for 1 h at room temperature and incubated with primary antibodies overnight at 4°C. The blots were finally detected using a two-color infrared imaging system (Odyssey; LICOR) after incubation with the corresponding secondary antibody for 1 h. Protein levels were quantified and normalized to the GAPDH protein.

### Histological Analysis

The aortic tissues were fixed in 10% formalin and then embedded in paraffin. The samples were next sectioned at a thickness of 4–5 μm before deparaffination and rehydration. To evaluate the morphology of the aortic tissues, the sections were stained with elastic Van Gieson (EVG) staining.

### Immunohistochemistry and Immunofluorescence Analysis

For immunohistochemistry, the sections were blocked with 10% goat serum and incubated with the antibodies for anti-ADAMTS-5 (ab41037, Abcam), overnight at 4°C. Then, the sections were incubated with the appropriate secondary antibodies, and the nuclei were stained with DAPI solution at room temperature. Finally, the sections were visualized with diaminobenzidine (DAB) (Gene Tech, Shanghai, China) and viewed under a light microscope (Nikon H550L, Tokyo, Japan).

For immunofluorescence analysis, the sections were blocked with 10% goat serum and incubated with anti-ADAMTS-5 (Abcam), anti-CD31 (R&D Systems), anti-CD68 (R&D Systems), and anti-α-smooth muscle cell (α-SMA, GeneTex) antibodies overnight at 4°C. Then, the sections were incubated with two different IRDye® 800CW-conjugated 169 secondary antibodies. Finally, fluorescence images were captured with an OLYMPUS DX51 fluorescence microscope.

### Collection of Human Blood Samples

From October 2016 to March 2018, consecutive patients who suffered from sudden chest pain and were hospitalized in the People's Hospital of the Guangxi Zhuang Autonomous Region were enrolled in this study. The exclusion criteria for the discovery set were as follows: (a) receiving blood transfusions before collecting blood; (b) aortic trauma, peripheral arterial disease, or pseudoaneurysm; (c) a history of coronary heart disease, valvular heart disease, or heart failure; (d) severe hepatic or renal dysfunction; and (e) autoimmune diseases, infectious diseases, or active cancer. After computed tomography angiography (CTA) of the aorta was performed, the patients returned to the ICU, and blood samples were immediately collected in vacutainer tubes containing sodium heparin. All plasma samples were centrifuged at 4,000 g for 20 min, and the supernatants were collected and stored at −80 °C. According to the CTA results, clinical features, and diagnostic clinical results of the patients, they were divided into a non-AD group (NAD, *n* = 41) and an AAD group (*n* = 83). The patients in the AAD group were further divided into Stanford A (*n* = 37), and Stanford B groups (*n* = 46). The patients and their families provided informed consent for donation. This study procedure was approved by the Medical Ethics Committee of the People's Hospital of the Guangxi Zhuang Autonomous Region.

### Blood Sample Measurement

The blood samples above were removed from storage at −80°C and thawed at room temperature. The plasma levels of ADAMTS-5 and matrix metalloproteinase (MMP)-2 and MMP-9 were measured with enzyme-linked immune sorbent assay (ELISA) kits from R&D Systems, while interleukin (IL)-1β was measured using ELISA kits from eBioscience.

### Cell Culture and Treatment

Human aortic smooth muscle cells (HASMCs) (Cell Applications, USA) were cultured in the DMEM/F12 medium containing 10% fetal bovine serum (FBS) and penicillin (100 U/mL)/streptomycin (100 μg/mL) in a humidified 5% CO_2_ atmosphere, with incubation at 37°C. Then, HASMCs were pretreated with recombinant human ADMATS5 (rhADAMTS-5, 5 μg/mL) for 6 h, followed by stimulation with 1 μmol/L of angiotensin II (AngII) for 12 h. In addition, terminal deoxynucleotidyl transferase dUTP nick-end labeling (TUNEL) staining was performed to measure HASMC apoptosis according to the instructions of the reagent manufacturer (Roche, Germany).

### RT-PCR

Total RNA from HASMCs was extracted with the TRIzol reagent (Roche, Germany). Then, cDNA was synthesized from 2 μg of RNA using oligo (DT) primers and the Transcriptor First Strand cDNA Synthesis Kit (Roche, Germany). RT-PCR was carried out using a LightCycler 480 and SYBR Green Master Mix (Roche, Germany) according to the manufacturer's instructions. The results were quantified and normalized against GAPDH expression. The details of the primers are presented in Table 1.

**Table 1:** Primer sequences for RT-PCR assays.

**Gene**		**Sequence (5′-3′)**
MMP-2	Forward	CTACGACCGCGACAAGAAGT
	Reverse	AGTTCCCACCAACAGTGGAC
MMP-9	Forward	GTACCACGGCCAACTACGAC
	Reverse	GCCGTCCTGGGTGTAGAGT
TNF-α	Forward	CCTCTCTCTAATCAGCCCTCTG
	Reverse	GAGGACCTGGGAGTAGATGAG
Bax	Forward	ATGGACGGGTCCGGGGAGCAGCCC
	Reverse	GGTGAGCACTCCCGCCACAAAGAT
Bcl-2	Forward	AAGAGCAGACGGATGGAAAAAGG
	Reverse	GGGCAAAGAAATGCAAGTGAATG
GAPDH	Forward	TCCACTGGCGTCTTCACC
	Reverse	GGCAGAGATGATGACCCTTTT

### Statistical Analysis

Plasma cytokine concentrations and clinical characteristics were expressed as medians (lower quartile to upper quartile) and compared with Mann–Whitney U tests. The categorical variables were defined as percentages and compared using the chi-square test. Spearman's correlation was used to calculate the correlations between plasma ADAMTS-5, MMP-2, MMP-9, and TNF-α and clinical characteristics. To identify independent predictors of the occurrence of AAD, simple linear regression analyses and subsequent multivariate linear regression analyses were performed. A two-tailed *P* < 0.05 was considered statistically significant.

## Results

### Basic Clinical Characteristics of Patients Who Provided Aortic Tissue Samples

Among the patients who provided aortic tissue samples, the white blood cell count (WBC) and the levels of C-reactive protein (CRP) and D-dimer were higher in the AAD patients. No differences were found between the two groups for other clinical characteristics, including sex, age, smoking, glucose (Glu), triglycerides (TG), total cholesterol (TC), high-density lipoprotein cholesterol (HDL-C), low-density lipoprotein cholesterol (LDL-C), and creatinine (CREA). The clinical data for all patients are listed in Table 2.

**Table 2:** Clinical Characteristics in patients who provide aortic tissue samples.

**Characteristics**	**Control**	**AAD**	***P***
Gender (M/F)	4/3	7/2	0.377
Age (years)	44 (37, 61)	57 (40, 67)	0.337
Smoking (*n*, %)	2 (28.6%)	5 (55.6%)	0.398
Glu (mmol/L)	5.3 (4.8, 6.1)	5.2 (4.6, 5.7)	0.535
TG (mmol/L)	1.21 (0.99, 1.68)	0.98 (0.82, 1.98)	0.312
TC (mmol/L)	4.31 (3.95, 4.94)	4.07 (3.76, 4.56)	0.365
HDL (mmol/L)	1.88 (1.19, 2.14)	1.75 (1.28, 2.15)	0.962
LDL (mmol/L)	1.81 (1.52, 2.66)	2.51 (1.74, 3.01)	0.396
SBP (mmHg)	-	154 (140, 160)	-
DBP (mmHg)	-	87 (79, 96)	-
CREA (μmol/L)	68 (58, 74)	74 (61, 90)	0.358
WBC (×10^9^/L)	5.76 (4.46, 6.25)	14.37 (8.75, 17.45)	0.000
CRP (mg/L)	0.62 (0.25, 2.04)	5.74 (2.80, 14.5)	0.011
D-dimer (μg/mL)	0.86 (0.71, 0.96)	6.96 (3.27, 10.32)	0.000

*Glu, fasting glucose; TG, total triglycerides; TC, total cholesterol; HDL–C, high-density lipoprotein cholesterol; HDL–C, low-density lipoprotein cholesterol; SBP, systolic blood pressure; DBP, diastolic blood pressure; CREA, creatinine; WBC, white blood cell; CRP, C-reactive protein*.

### Expression of ADAMTS-5 in Human Thoracic Aortic Tissue

**Figure 1:**
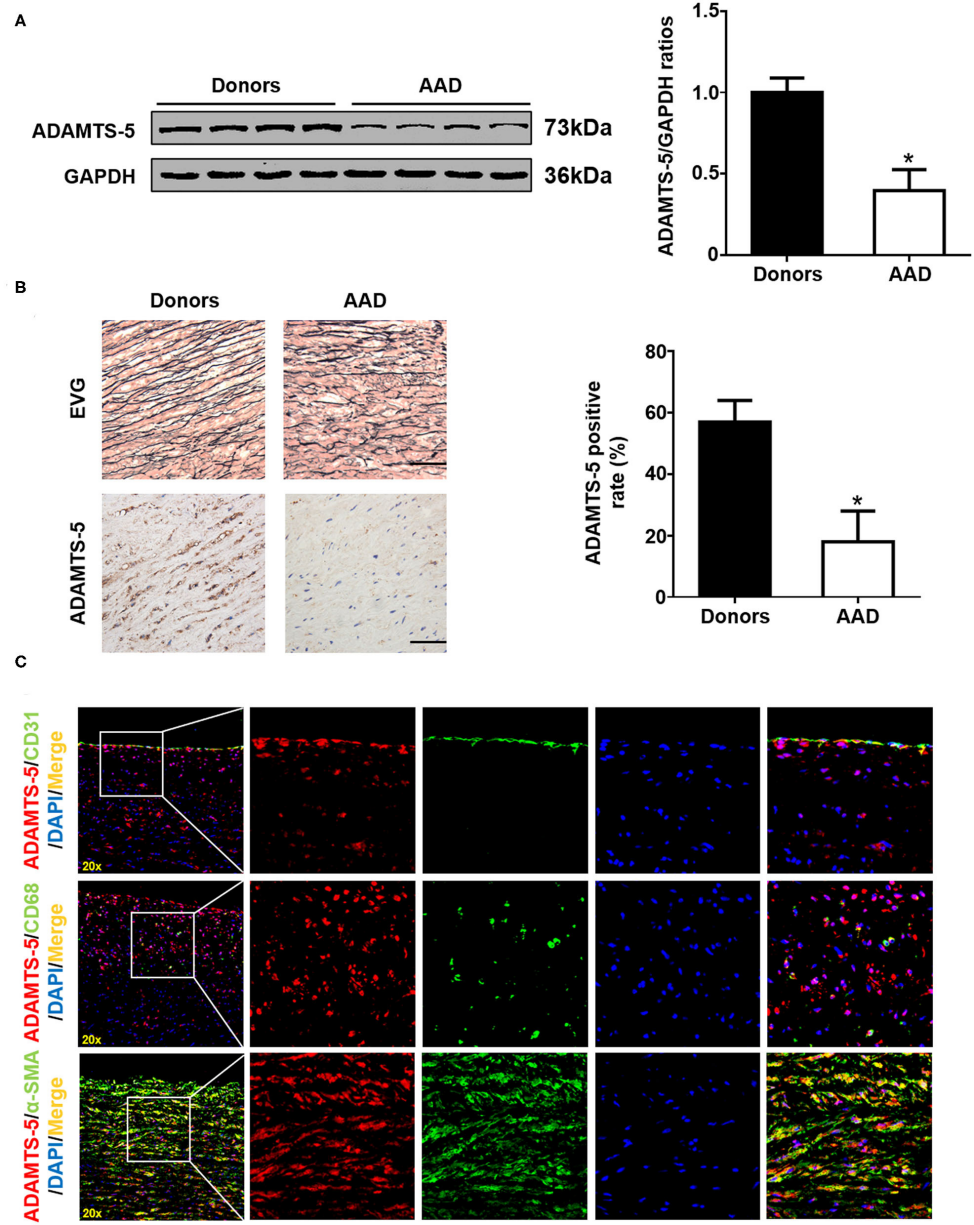
ADAMTS-5 expression in human aortic tissues. **(A)** The levels of ADAMTS-5 in aortic tissues from normal donors and AAD patients were detected by western blotting. *P < 0.05 vs. donors. **(B)** Representative images of elastic Van Gieson (EVG) staining and immunohistochemistry staining of human aortic tissues from normal donors or acute aortic dissection (AAD) patients. Scale bar, 50 μm. **(C)** The source of ADAMTS-5 in aortic tissues from AAD patients was detected by double-immunofluorescence staining.

ADAMTS-5 expression in AAD was measured via western blot and histopathological analyses, and the results showed that decreased ADAMTS-5 levels occurred in the aortas of AAD patients (Figures 1A,B). In addition, double-immunofluorescence staining indicated that VSMCs were the main source of ADAMTS-5 (Figure 1C).

### Baseline Characteristics of Patients Who Provide Blood Samples

Among the patients who provided blood samples, the incidence rate of uncontrolled blood pressure (HBP) and the levels of SBP, DBP, Glu, WBC, CRP, and D-dimer were significantly increased in the AAD patients. However, other clinical characteristics, including age, sex, smoking, heart rate (HR), TG, TC, HDL-C, and LDL-C and the administration of therapeutic agents, were not significantly different between the two groups. There were no significant differences between the Stanford A and Stanford B patients in terms of clinical characteristics. The baseline clinical characteristics of the four groups are presented in Table 3.

**Table 3:** Clinical characteristics of patients who provided peripheral blood.

**Characteristics**	**NAD**	**AAD**	**Type A**	**Type B**
Age (years)	60 (46, 65)	62 (51, 67)	58 (52, 65)	62 (48, 67)
Gender (M/F)	30/11	66/17	29/8	37/9
Smoking (*n*, %)	13 (31.7)	37 (44.5)	18 (48.6)	19 (41.3)
HBP (*n*, %)	16 (39.0)	58 (69.8) ^*^	28 (75.6) ^*^	30 (65.2) ^*^
HR (bpm)	24.3 (22.4, 26.9)	24.5 (22.7, 27.1)	24.1 (22.4, 27.1)	24.6 (22.6, 27.2)
SBP (mmHg)	129 (113, 145)	145 (131, 169)	150 (139, 170) ^*^	145 (130, 168) ^*^
DBP (mmHg)	74 (65, 83)	80 (70, 97) ^*^	80 (72, 95) ^*^	80 (70, 97) ^*^
WBC (×10^9^/l)	6.2 (5.0, 7.6)	11.7 (8.7, 14.4) ^*^	11.9 (9.1, 14.8) ^*^	11.0 (8.0, 13.9) ^*^
TC (mmol/l)	4.0 (3.2, 5.5)	4.2 (3.4, 5.7)	4.1 (3.6, 5.8)	4.3 (3.3, 5.7)
TG (mmol/l)	1.3 (1.0, 1.8)	1.2 (0.9, 2.0)	1.2 (0.8, 1.9)	1.2 (0.9, 2.0)
HDL-C (mmol/l)	1.0 (0.8, 1.2)	1.1 (0.7, 1.4)	1.0 (0.8, 1.4)	1.1 (0.7, 1.4)
LDL-C (mmol/l)	2.1 (1.5, 2.5)	2.2 (1.6, 2.8)	2.2 (1.7, 2.6)	2.4 (1.6, 2.8)
Glu (mmol/l)	6.0 (5.3, 6.9)	7.3 (6.4, 8.5) ^*^	7.3 (6.6, 8.2) ^*^	7.1 (6.1, 8.9) ^*^
CREA (μmol/l)	79 (68, 87)	87 (71, 110) ^*^	89 (71, 114) ^*^	86 (74, 109) ^*^
D-dimer (μg/ml)	0.8 (0.3, 1.3)	4.0 (2.1, 6.6) ^*^	3.3 (2.0, 6.3) ^*^	4.3 (2.2, 6.8) ^*^
CRP (mg/l)	1.0 (0.4, 2.2)	16.4 (3.8, 71.2) ^*^	18.4 (5.3, 66.1) ^*^	15.8 (3.7, 77.6) ^*^
Medications, *n* (%)				
ACEI/ARB	27 (65.8%)	45 (54.2%)	21 (56.7%)	24 (48.9%)
β blockers	14 (34.1%)	30 (35.7%)	9 (24.3%)	12 (26.1%)
CCB	24 (58.5%)	34 (41.0%)	16 (43.2%)	18 (39.1%)
Diuretics	3 (7.3%)	8 (9.64%)	3 (8.1%)	5 (12.2%)

*HBP, uncontrolled or failure to control of blood pressure; HR, heart rate; SBP, systolic blood pressure; DBP, diastolic blood pressure; WBC, white blood cell; TC, total cholesterol; TG, total triglycerides; HDL-C, high-density lipoprotein cholesterol; LDL-C, low-density lipoprotein cholesterol; Glu, fasting glucose; CREA, creatinine; CRP, C-reactive protein; ACEI, angiotensin-converting enzyme inhibitor; ARB, angiotensin receptor blocker; CCB, calcium channel blocker*.

* *p < 0.05 vs. NAD group*.

### Plasma Cytokine Concentrations in AAD Patients

**Figure 2:**
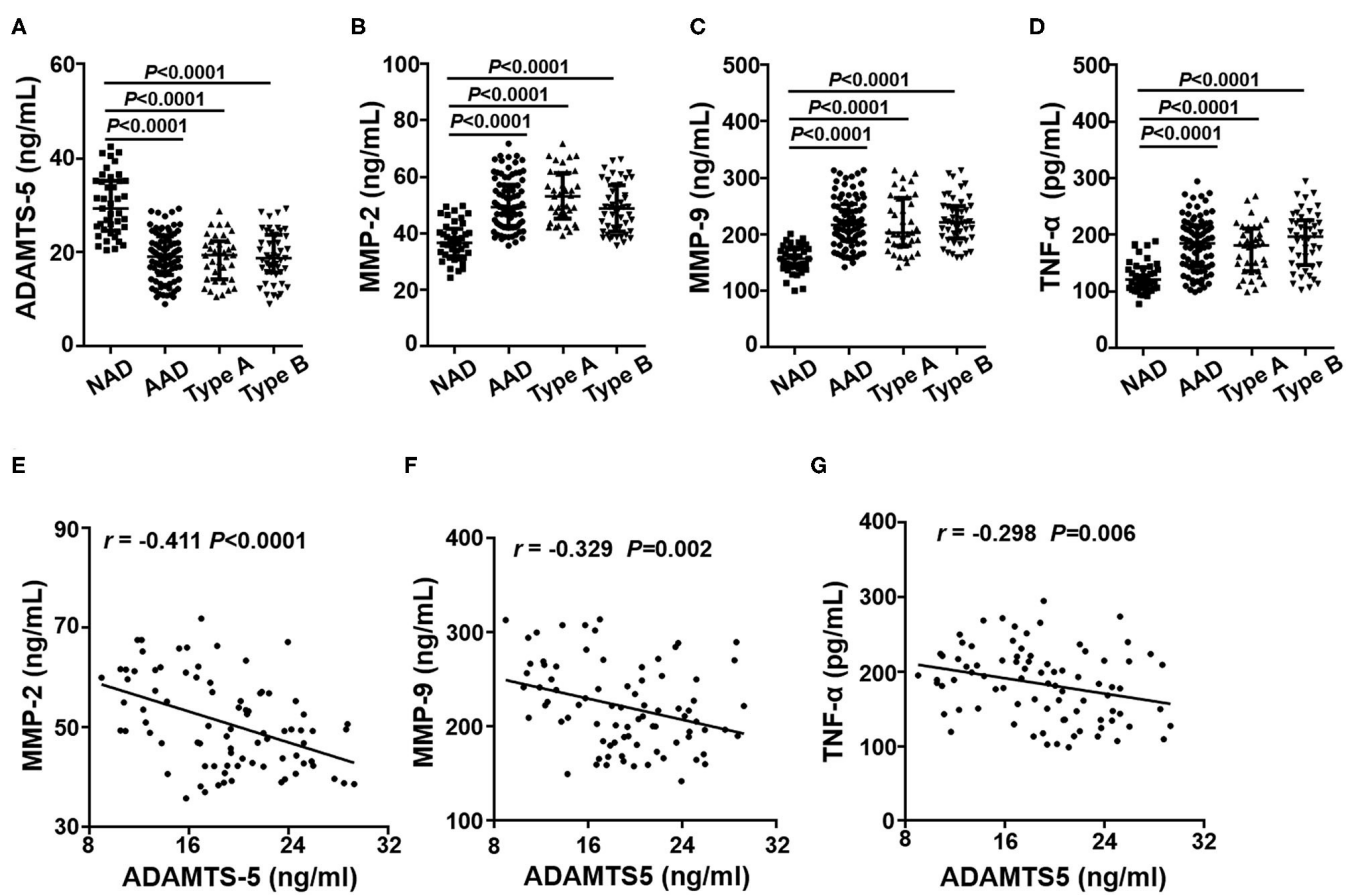
Plasma cytokine levels in each group. The plasma levels of ADAMTS-5 **(A)**, MMP-2 **(B)**, MMP-9 **(C)**, and TNF-α **(D)** in the NAD and AAD groups were measured by ELISA. Spearman's correlation between plasma levels of MMP-2 **(E)**, MMP-9 **(F)**, TNF-α **(G)**, and ADAMTS-5 in AAD patients.

Plasma concentrations of these cytokines were detected by ELISA, and the results showed that plasma ADAMTS-5 levels were lower in the AAD group than in the NAD group (Figure 2A). Plasma MMP-2, MMP-9, and TNF-α levels, by contrast, were even higher in AAD patients (Figures 2B–D). In addition, there were no significant differences between the Stanford A and Stanford B groups (Figures 2A–D). The plasma measured in each group is shown in Table 4. Furthermore, Spearman's correlation analysis indicated that plasma ADAMTS-5 levels were negatively correlated with MMP-2 levels (r = −0.411, *P* < 0.0001), MMP-9 levels (r = −0.329, *P* = 0.002), and TNF-α levels (r = −0.298, *P* = 0.006) (Figures 2E–G).

**Table 4:** Plasma cytokines in NAD and AAD group.

**Characteristics**	**NAD**	**AAD**	**Type A**	**Type B**
ADAMTS-5 (ng/mL)	29.4 (24.6, 35.2)	19.1 (15.2, 23.4) ^*^	19.4 (14.3, 22.4) ^*^	18.9 (15.6, 23.8) ^*^
MMP-2 (ng/mL)	36.7 (32.0, 41.6)	49.3 (42.7, 57.3) ^*^	53.2 (45.2, 61.4) ^*^	48.8 (40.7, 57.1) ^*^
MMP-9 (ng/mL)	156.6 (140.8, 175.1)	216.9 (188.8, 253.8) ^*^	202.8 (180.1, 264.3) ^*^	221.1 (193.3, 250.1) ^*^
TNF-α (pg/mL)	120.6 (104.1, 142.1)	184.9 (143.6, 216.8) ^*^	181.2 (135.6, 210.9) ^*^	196.9 (146.4, 225.9) ^*^

* *p < 0.05 vs. NAD group*.

### Correlation Between Plasma Cytokine Concentrations and Clinical Characteristics

**Figure 3:**
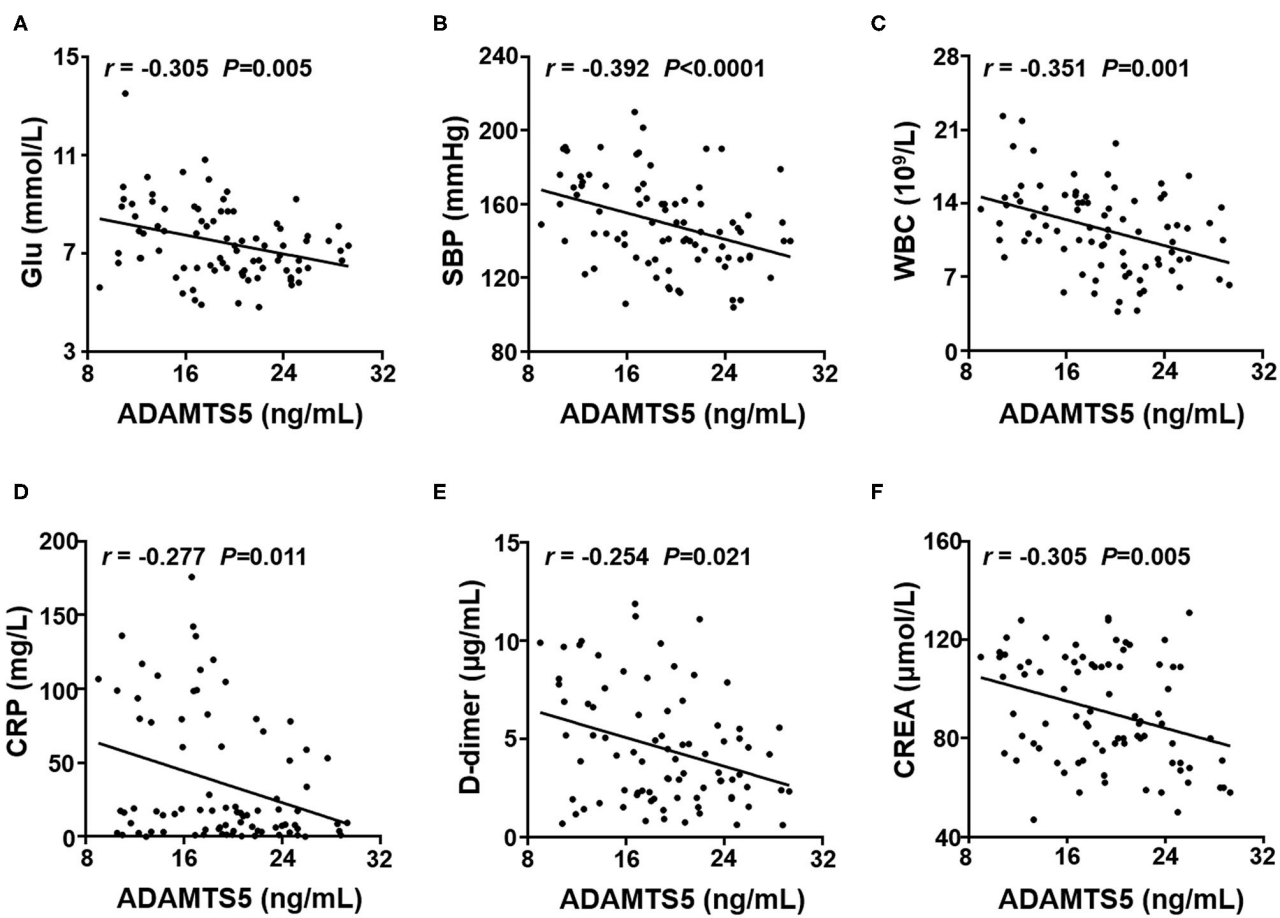
Correlation between plasma ADAMTS-5 levels and clinical characteristics. Correlations between ADAMTS-5 levels and **(A)** Glu, **(B)** SBP, **(C)** WBC, **(D)** CRP, **(E)** D-dimer, and **(F)** CREA. Glu, fasting glucose; SBP, systolic blood pressure; WBC, white blood cell; CRP, C-reactive protein; CREA, creatinine.

To further explore whether the plasma ADAMTS-5 concentration was associated with clinical characteristics, the Spearman's correlations between ADAMTS-5 and clinical characteristics were analyzed. The results showed that plasma ADAMTS-5 levels were also negatively correlated with Glu (r = −0.305, *P* = 0.005), SBP (r = −0.392, *P* < 0.0001), WBC (r = −0.351, *P* = 0.001), CRP (r = −0.277, *P* = 0.011), D-dimer (r = −0.254, *P* = 0.021), and CREA (r = −0.305, *P* < 0.005) (Figures 3A–F).

### Simple Linear Regression Analysis and Multivariate Linear Regression Analysis

To determine independent predictors of the presence of AAD, simple linear regression analyses and subsequent multivariate linear regression analyses were performed. Simple linear regression analyses showed that ADAMTS-5, MMP-2, MMP-9, and TNF-α levels and HBP, Glu, WBC, CREA, CRP, and D-dimer levels exhibited a trend toward an association with the presence of AAD, whereas sex, age, HR, smoking, TC, TG, HDL, and LDL showed no obvious trend toward such an association. Furthermore, multivariate linear regression analyses were performed. The results demonstrated that ADAMTS-5 was independently associated with the presence of AAD. MMP-2, MMP-9, TNF-α, HBP, and D-dimer were also associated with the presence of AAD (Table 5).

**Table 5:** Association between cytokines, clinical characteristics and the presence of AAD were assessed by simple linear regression analysis and subsequent binary linear regression analysis.

**Variables**	**Simple linear**			**Binary linear**		
	**β**	**95% CI**	***P*-value**	**β**	**95% CI**	***P*-value**
ADAMTS-5	−0.686	−0.816 to −0.556	0.000	−0.346	−0.036 to −0.007	0.005
MMP-2	0.619	0.478 to 0.760	0.000	0.193	0.000 to 0.003	0.007
MMP-9	0.609	0.466 to 0.751	0.000	0.176	0.002 to 0.014	0.014
TNF-α	0.542	0.392 to 0.693	0.000	0.164	0.000 to 0.003	0.013
HBP	0.299	0.126 to 0.468	0.001	−0.200	−0.352 to −0.032	0.019
Glu	0.349	0.180 to 0.516	0.000	0.048	−0.021 to 0.049	0.427
WBC	0.583	0.437 to 0.728	0.000	0.113	−0.003 to 0.028	0.118
CREA	0.291	0.119 to 0.462	0.001	0.009	−0.002 to 0.003	0.878
CRP	0.417	0.254 to 0.580	0.000	0.056	−0.001 to 0.002	0.367
D-dimer	0.582	0.436 to 0.728	0.000	0.152	0.003 to 0.045	0.027
Gender	0.071	−0.107 to 0.250	0.431			
Age	0.175	−0.001 to 0.352	0.052			
HR	0.023	−0.156 to 0.203	0.796			
Smoking	0.146	−0.032 to 0.323	0.106			
TC	0.038	−0.141 to 0.217	0.678			
TG	0.031	−0.148 to 0.210	0.734			
HDL	0.030	−0.149 to 0.209	0.742			
LDL	0.124	−0.054 to 0.302	0.169			

### ADAMTS-5 Ameliorates Ang II-Induced HASMC Apoptosis

**Figure 4:**
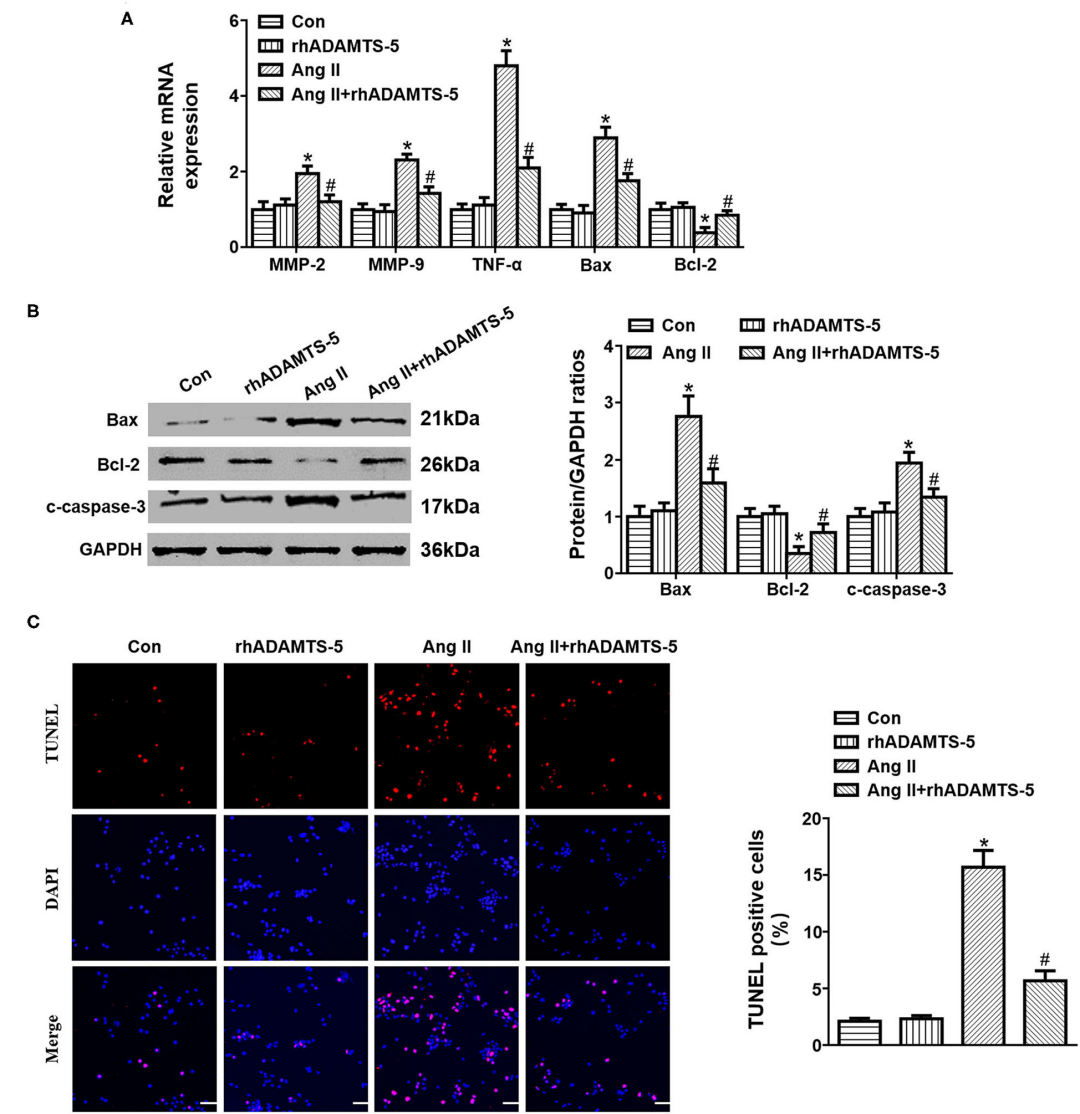
Effect of ADAMTS-5 treatment on angiotensin II (Ang II)-induced SMC apoptosis *in vitro*. **(A)** RT-PCR analyses of MMP-2, MMP-9, TNF-α, Bax, and Bcl-2 mRNA and protein expression *in vitro*. **(B)** TUNEL staining and quantitation analysis in the indicated groups. *P < 0.05 vs. control. ^#^*P* < 0.05 vs. Ang II. **(C)** Western blotting analyses of Bax, Bcl-2 and c-caspase 3 protein expression *in vitro*.

To investigate the role of ADAMTS-5 in regulating SMC apoptosis, we cultured HASMCs under Ang II stimulation and analyzed relative cytokine expression. The RT-PCR and western blotting results revealed that Ang II treatment could induce HASMC apoptosis by increasing the expression of Bax and c-caspase-3 and decreasing the expression of Bcl-2 (Figures 4A,B). In addition, Ang II treatment increased the expression of MMP-2, MMP-9, and TNF-α, whereas rhADMATS5 decreased the expression of MMP-2, MMP-9, and TNF-α (Figures 4A,B). The TUNEL staining results further revealed that rhADMATS5 treatment decreased Ang II-induced HASMC apoptosis (Figure 4C).

## Discussion

In this study, we demonstrated that the ADAMTS-5 protein was significantly decreased in AAD aortas and that SMCs were the main source of ADAMTS-5 in AAD aortas. In addition, the plasma ADAMTS-5 concentration was decreased, and the levels of the AAD-associated cytokines MMP-2, MMP-9, and TNF-α were dramatically increased in AAD patients. Furthermore, the plasma ADAMTS-5 level was negatively correlated with these cytokines, and the decreased ADAMTS-5 level was independently associated with the occurrence of AAD. More importantly, rhADMATS5 ameliorated Ang II-induced SMC apoptosis.

ADAMTS-5, an extracellular metalloproteinase, is a recently identified member of the ADAMTS family that has been shown to participate in ECM remodeling. Moreover, ADAMTS-5 seems to exhibit the highest aggrecanase activity among ADAMTS family proteins and to be responsible for proteoglycan cleavage (14). ADAMTS-5 is a secreted protease that is involved in various pathophysiological processes, such as osteoarthritis, intervertebral disc degeneration, cancer, and angiogenesis (10, 15–17). Echtermeyer et al. reported that a lack of ADAMTS-5 protected against cartilage degradation in a murine model of osteoarthritis (15). Additionally, decreased ADAMTS-5 expression was observed in prostate cancer and coincided with the accumulation of versican (18).

More recently, ADAMTS-5 has been reported to be involved in vascular ECM turnover and vascular diseases. In a controversial report about ADAMTS-5 and thoracic aortic aneurysms and dissections, Mahmut et al. reported that ADAMTS5 protein expression was significantly higher in thoracic aortic aneurysm and dissection tissues than in control aortic tissues (19). By contrast, Cikach et al. reported that ADAMTS-5 mRNA expression was decreased while aggrecan and versican were increased in AAD aortas compared with control aortas (13). In the present study, we found that ADAMTS-5 expression was markedly decreased in both the aortas and plasma of AAD patients. In addition, multivariate linear regression analyses showed that ADAMTS-5 was independently associated with the presence of AAD. The results showed that ADAMTS-5 may participate in the pathogenesis and progression of AAD.

It is well established that ECM homeostasis may affect intrinsic properties and stiffness of the arterial wall and thereby increase susceptibility to AAD. Metalloproteinases, including MMPs, ADAMs, and ADAMTSs, have been shown to play a critical role in degradation of the ECM (20, 21). MMPs, especially MMP-2 and MMP-9, are responsible for ECM remodeling and the development of AAD (21–24). Kurihara et al. reported that the plasma MMP-9 level was significantly elevated in AAD patients compared with patients with acute myocardial infarction or healthy volunteers (22). Ishii et al. found that the expression of MMP-2 was significantly higher during the granulation tissue response after the onset of AAD (23). Consistent with previous research, our results showed that plasma levels of MMP-2 and MMP-9 were markedly higher in the AAD group than in the NAD group. Furthermore, Spearman's correlation analysis indicated that plasma ADAMTS-5 levels were negatively correlated with MMP-2 and MMP-9 levels. These results showed that decreases in ADAMTS-5 and increases in MMP-2 and MMP-9 in SMCs accelerate ECM remodeling and thereby contribute to medial degeneration and AAD.

Mounting evidence demonstrates that immune-inflammatory responses contribute greatly to the progress of AAD (25–29). As a proinflammatory cytokine, TNF-α is known as the gatekeeper of inflammation and a mediator of the inflammatory cascade (30, 31). Kimura et al. demonstrated that TNF-α mRNA expression was significantly higher in AAD aortic tissues than in those from normal donors (32). Additionally, previous studies showed that TNF-α plays a critical role in ECM degradation and ADAMTS-5 activation in articular cartilage (33, 34). Consistent with previous results, we found that plasma TNF-α was dramatically increased in AAD patients. Furthermore, Spearman's correlation analysis showed that ADAMTS-5 levels were negatively associated with TNF-α. A possible explanation for this finding is that ADAMTS-5 may promote the association of AAD with secretion of inflammatory factors.

As SMCs are the major component of vascular systems, homeostasis of SMCs plays a crucial role in the progression of AAD (35). It is generally accepted that SMCs contribute to MMP synthesis and matrix protein proliferation, thereby maintaining ECM stabilization (8, 36). Accumulating data support the concept that AAD is associated with degeneration of the aortic media, characterized by loss of SMCs and ECM remodeling. Therefore, excessive loss of SMCs in the aorta is considered to be an important molecular mechanism in AAD development. In addition, an excessive inflammatory response also contributes to the apoptosis of SMCs and thereby causes excessive loss of SMCs. Suna et al. reported that the ADAMTS-5 protein was expressed in coronary artery SMCs and that its expression was notably reduced in a porcine model of stent injury (37). In our study, double-immunofluorescence staining indicated that ADAMTS-5 was expressed in SMCs, endothelial cells and macrophages, while aortic SMCs were the main source of ADAMTS-5. Furthermore, we found that ADAMTS-5 administration counteracted SMC apoptosis. Therefore, these results demonstrated that ADAMTS-5 serves as a therapeutic agent for AAD by inhibiting SMC apoptosis.

As an ECM-degrading proteinase, ADAMTS-5 is involved in ECM remodeling (10). However, it is still unclear for the concrete mechanism by which ADAMTS-5 exerts its biological effect. Previous researches has shown that low-density lipoprotein receptor-related protein 1 (LRP1) is a large endocytic receptor that is responsible for cellular uptake of ADAMTS-5 from the extracellular matrix (38, 39). In addition, LRP1 has also been found to modulate signaling pathways and to be involved in different cellular processes, including lipid homeostasis, signal transduction, and cell proliferation (40, 41). Fava et al. also reported that LRP1 was profoundly reduced in aortas of mice lacking the catalytic domain of ADAMTS-5. Moreover, silencing LRP1 in SMCs also reduced the expression of ADAMTS-5 and attenuated ADAMTS-5-mediated versican cleavage (42). Based on these reports, we speculate that LRP1 plays an important role in the biological effects of ADAMTS-5.

Several limitations of the present study should be mentioned. First, considering that it is difficult to obtain normal aortic tissue, the normal aortic tissue used in our study was obtained from people who experienced brain death following a traffic accident or head trauma. However, we are unsure whether these situations affect the expression of ADAMTS-5 in the aorta. Second, despite the exclusion criteria, cardiovascular disease cannot be completely ruled out in the NAD group. Therefore, the results should be interpreted cautiously. Finally, we did not detect dynamic changes in plasma ADAMTS-5 levels in the AAD patients and did not follow-up patients to assess long-term mortality or prognosis. Thus, we need to conduct follow-up studies to detect the clinical outcomes of these patients.

## Data Availability Statement

All datasets generated for this study are included in the article/supplementary material.

## Ethics Statement

The studies involving human participants were reviewed and approved by Medical Ethics Committee of the People's Hospital of Guangxi Zhuang Autonomous Region. The patients/participants provided their written informed consent to participate in this study.

## Author Contributions

TZ and JG performed the study, analyzed the data, and wrote the manuscript. TZ, JG, YLiu, LS, ZL, YX, RX, LL, and ZY contributed to the acquisition of data and to manuscript preparation and revision. TZ, JG, YLin, and JY conceived the hypothesis and participated in the experimental design, data interpretation, and manuscript preparation and revision. All authors approved the final version of the manuscript.

## Conflict of Interest

The authors declare that the research was conducted in the absence of any commercial or financial relationships that could be construed as a potential conflict of interest.
